# Supplementary material for: Pathways, Processes, and Candidate Drugs Associated with a Hoxa Cluster-Dependency Model of Leukemia
Source: Cancers (Basel). 2019 Dec 17;11(12):2036. doi: 10.3390/cancers11122036 (PMC6966468; doi:10.3390/cancers11122036)
Supplement: Supplementary file 1 [file cancers-11-02036-s001.zip › cancers-650290-suppl-proof/cancers-650290-suppl-.docx]

Supplementary Materials: Pathways, Processes, and Candidate Drugs Associated with a *Hoxa* Cluster-Dependency Model of Leukemia

Laura M. Kettyle, Charles-Étienne Lebert-Ghali, Ivan V. Grishagin, Glenda J. Dickson, Paul G. O’Reilly, David A. Simpson, Janet J. Bijl, Ken I. Mills, Guy Sauvageau and Alexander Thompson


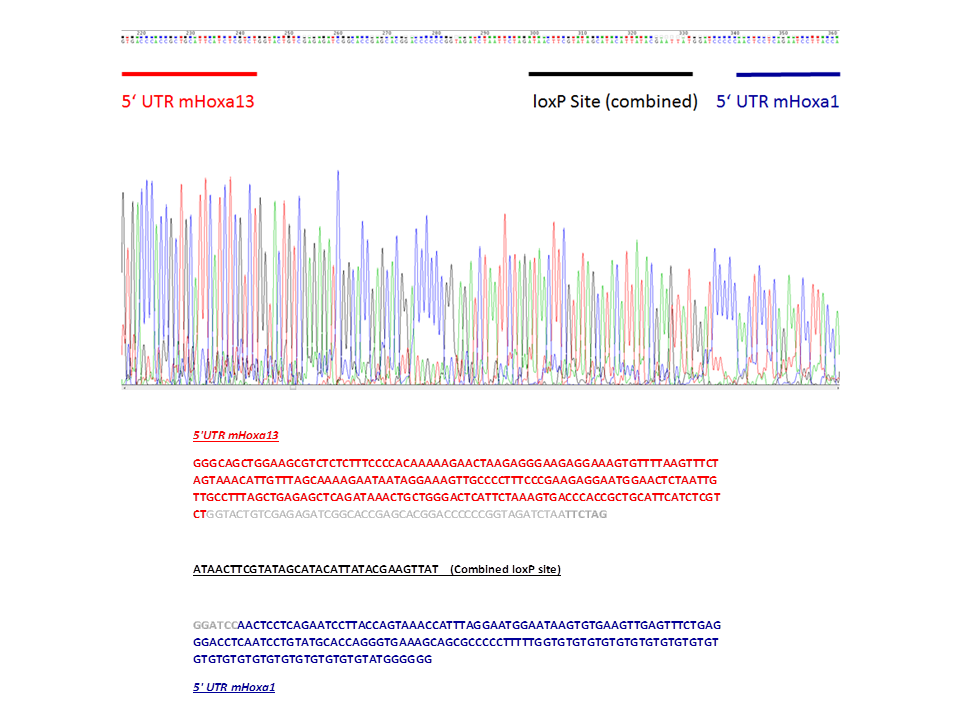


A

B


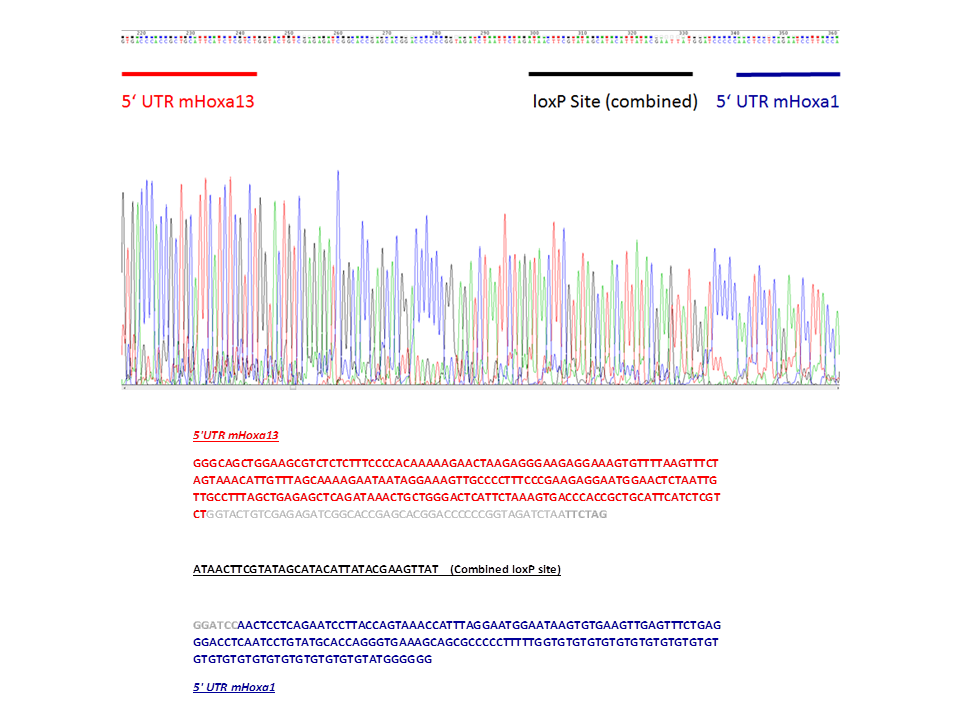


**3’ UTR *mHoxa1***

***loxP* site (combined)**

**5’ UTR *mHoxa13***

**Figure S1.** Direct sequence validation of the Hoxa^del^ amplicon. (**A**) Chromatogram of the central portion (~145 bp) of the *Hoxa*^del^ amplicon following Sanger sequencing. (**B**) A schematic of the complete sequence of the *Hoxa*^del^ amplicon obtained (~600 bp). Sequences aligned with the 5ʹ UTR of mHoxa13 (red), the combined loxP site (black) and 5ʹ UTR of mHoxa1 (blue) are annotated.


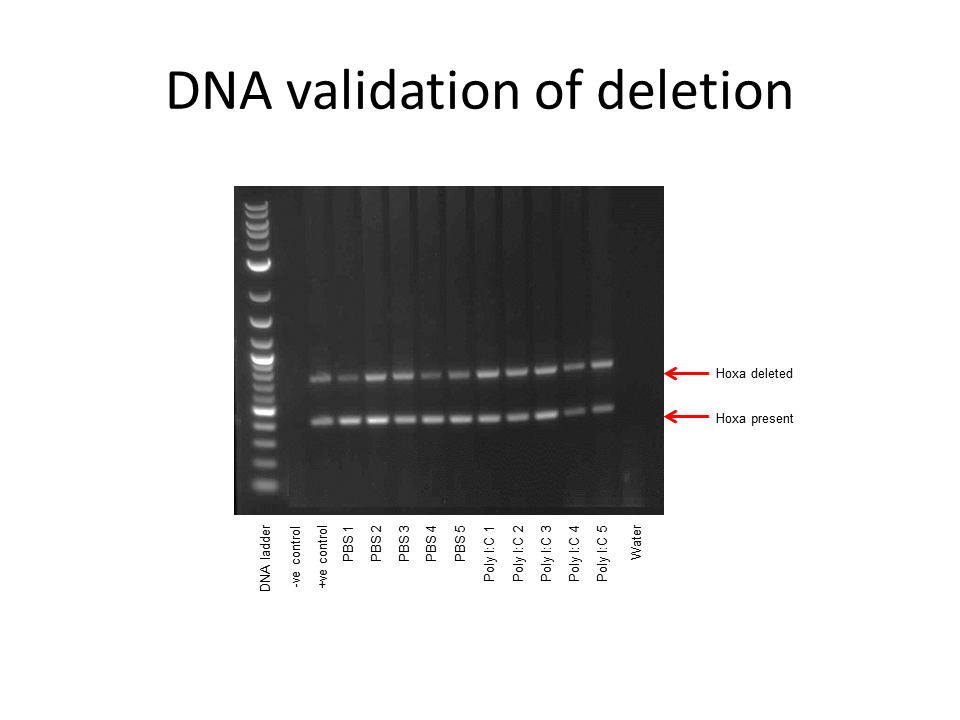


Hoxa^del^

Hoxa^wt/wt^

**Figure S2.** Retention of the Hoxa cluster in treated MAFF-MA9 leukemic mice. Digital images of an electrophoresed agarose gel containing PCR amplicons of expected size (arrows) derived from gDNA obtained from MAFF-MA9 derived leukemic mice bone marrow following necropsy. Transplanted cells were pre-treated with interferon-gamma and recipient mice were treated in vivo with Poly I:C or PBS (vehicle control) for up to 13 days (Figure 4A). No further deletion of the Hoxa cluster was observed following in vivo Poly I:C treatment (*n* = 5 for each group).


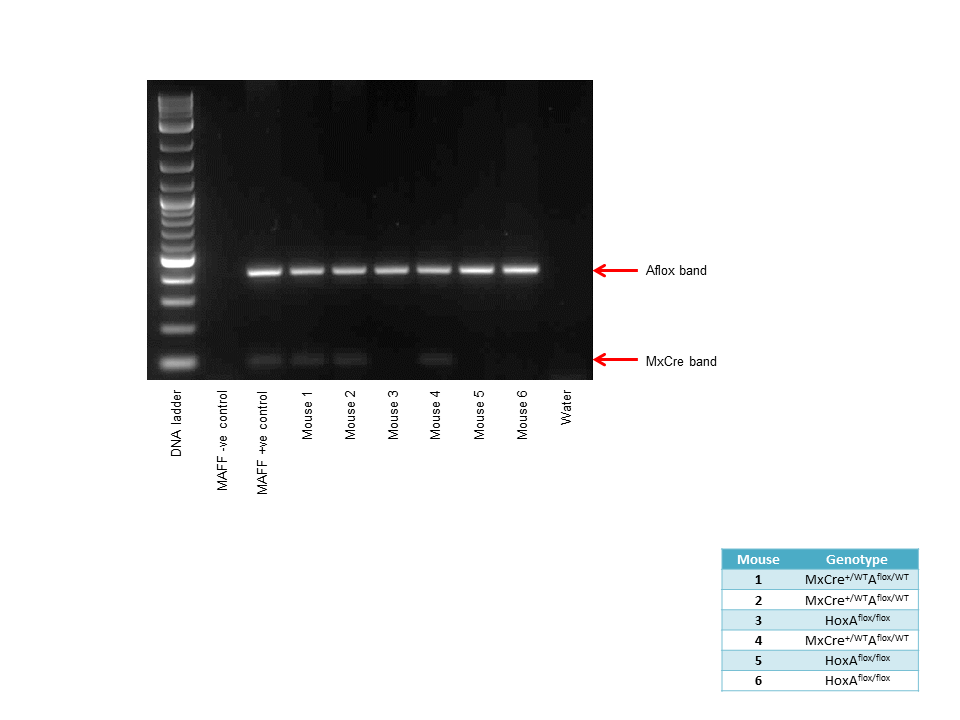

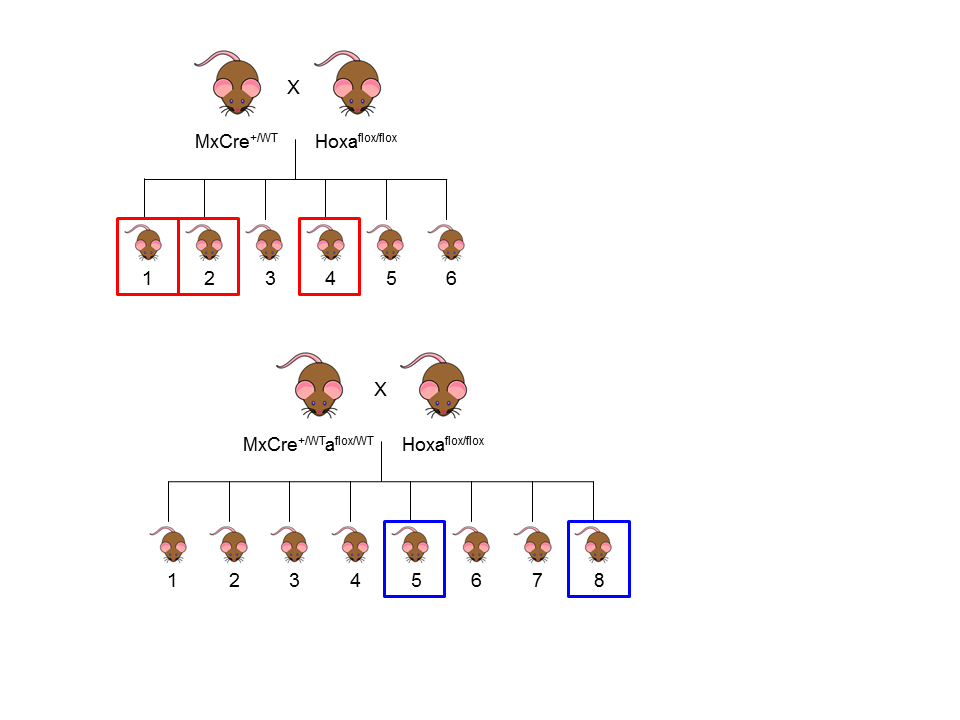


A


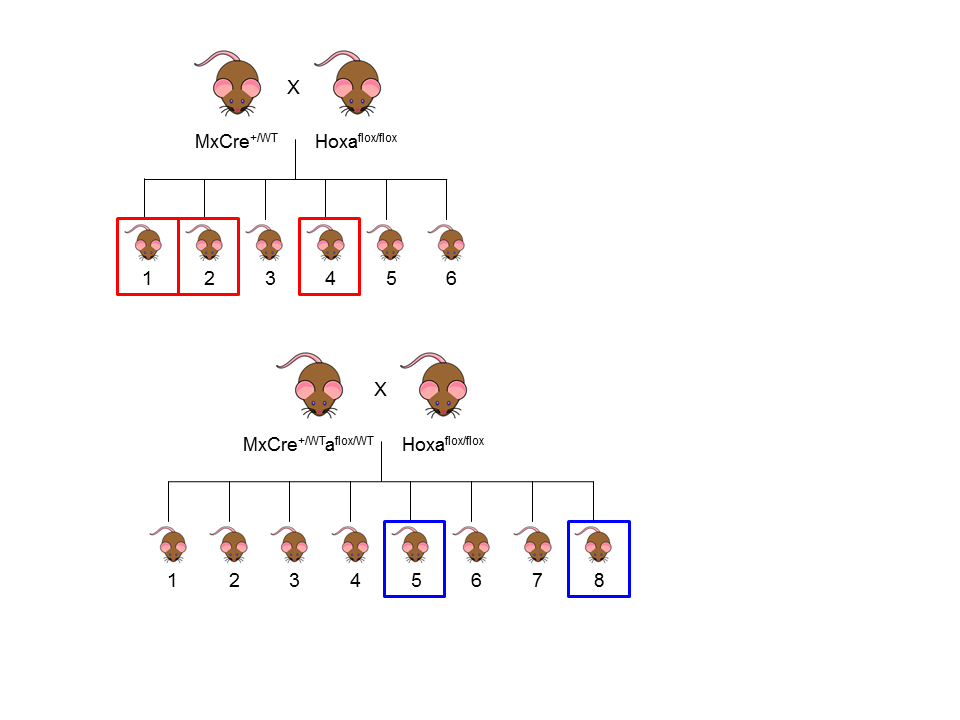


B


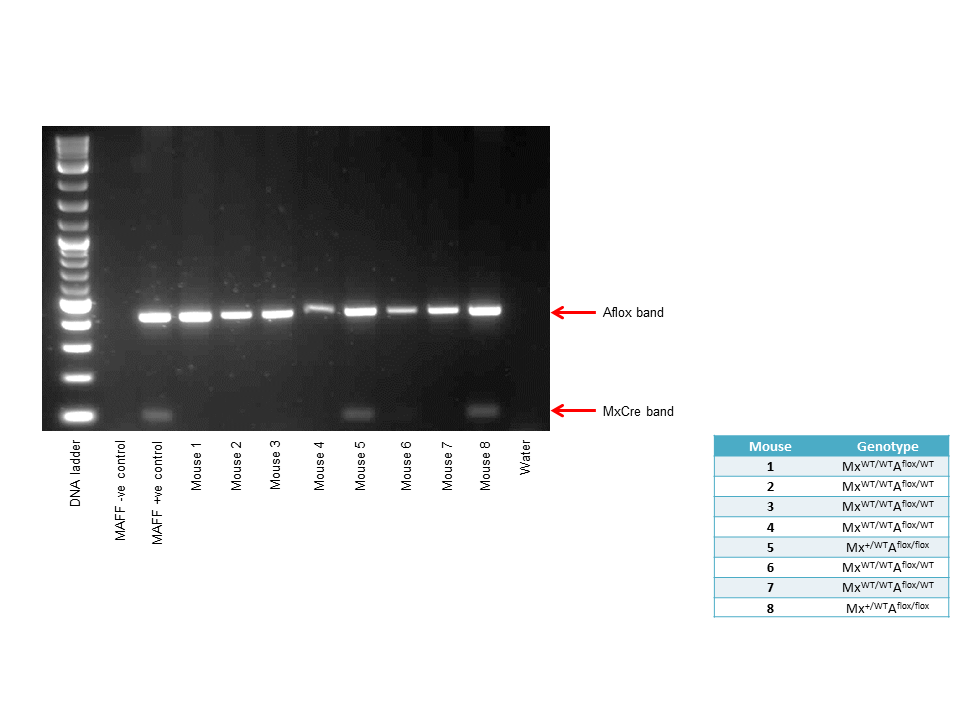


**Figure S3.** Genotyping of MxCre+/WTAflox/flox (MAFF) mice. Schematic of mouse breeding with associated sample digital images of electrophoresed agarose gels containing PCR amplicons of gDNA for genotyping. MxCre^+/WT^/Hoxa^flox/WT^ mice (**A**, red boxes) which were backcrossed with Hoxa^flox/flox^ mice to produce MxCre^+/WT^/Hoxa^flox/flox^ (MAFF) offspring (**B**, blue boxes) at normal Mendelian ratio.


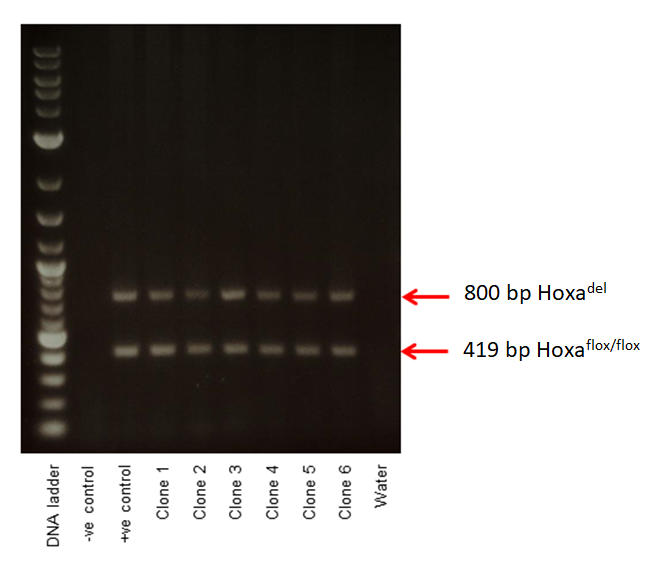


**Figure S4:** Sustained deletion of the Hoxa cluster in colonies derived from Cre-GFP leukemia cells. Digital image of an electrophoresed agarose gel containing PCR amplicons of gDNA for genotyping colonies generated from Cre-GFP treated MA9 leukemia cells grown as colonies for 10-14 days in methylcellulose.

**Table S1.** Representative Immunophenotype analysis of MLL-AF9 primary leukemias (PL) and cell lines (CL).

| **Sample Type** | **Gr-1^+^**  **Mac-1^-^ (%)** | **Gr-1^-^**  **Mac-1^+^ (%)** | **Gr-1^+^**  **Mac-1^+^ (%)** | **CD43^+^**  **IgM^-^ (%)** | **Lin^-^Sca^+^**  **Kit^-^ (%)** | **Lin^-^ Sca^-^**  **Kit^+^ (%)** | **Lin^-^ Sca^+^**  **Kit^+^ (%)** |
| --- | --- | --- | --- | --- | --- | --- | --- |
| **CD45.1 (PL)** | 27.9 | 0.1 | 2.2 | 89.6 | 2.6 | 26.3 | 0.9 |
| **CD45.1 (CL)** | 96.7 | 0 | 3.2 | 99.9 | 1.6 | 4.9 | 3.3 |
| **AFF (PL)** | 42.7 | 4.3 | 46.7 | 98.8 | 3 | 71.8 | 21.1 |
| **AFF (CL)** | 27.9 | 0 | 72.1 | 98.8 | 0.9 | 84.6 | 6.3 |
| **MAFF (PL)** | 64.4 | 5.3 | 5.1 | 96.6 | 5.2 | 15.3 | 2.2 |
| **MAFF (CL)** | 84.7 | 0 | 15.3 | 97.2 | 2.4 | 62.6 | 14.6 |

**Table S2.** Please check the excel file.

**Table S3.** List of oligomers used to define the genotype of experimental mice.

| **Genotype** | **Primer Set** | **Annealing**  **Temp °C** | **Amplicon**  **Length (bp)** |
| --- | --- | --- | --- |
| **WT** | Forward: AAAACGGGGGAGGGGTGCGGGC  Reverse: GGGGTGGGGGGCAAAGAGATAA | 64 | 693 |
| **Aflox** | Forward: GGCGAAGAATCTCGTGCTTTC  Reverse: CGGTGTCGTCCATCACAGTTTG | 64 | 419 |
| **MxCre** | Forward: GCGGTCTGGCAGTAAAAACTATC  Reverse: GTGAAACAGCATTGCTGTCACTT | 57 | 100 |
| **Aflox^del^** | Forward: GTGACTTTCTTTCTTGACCTCCCAACTC  Reverse: ACCTGAGTGAATGGGCAGAAAAACTCGAGG | 60 | 800 |
| **Hygro** | Forward: TCAGCGAGAGCCTGACCTAT  Reverse: AGTTCGGTTTCAGGCAGGTC | 58 | 78 |
| **Del** | Forward: CTAAAGTGACCCACCGCTGC  Reverse: TGAGGAGTTGGGGGATCCATAA | 58 | 109 |

| 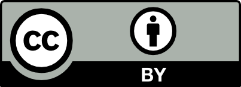 | © 2019 by the authors. Licensee MDPI, Basel, Switzerland. This article is an open access article distributed under the terms and conditions of the Creative Commons Attribution (CC BY) license (http://creativecommons.org/licenses/by/4.0/). |
| --- | --- |
